# To Tell or Not to Tell: Exploring the Preferences and Attitudes of Patients and Family Caregivers on Disclosure of a Cancer-Related Diagnosis and Prognosis

**DOI:** 10.1200/JGO.19.00132

**Published:** 2019-11-26

**Authors:** Arunangshu Ghoshal, Naveen Salins, Anuja Damani, Jayeeta Chowdhury, Arundhati Chitre, Mary Ann Muckaden, Jayita Deodhar, Rajendra Badwe

**Affiliations:** ^1^ Tata Memorial Centre, Mumbai, India; ^2^ Kasturba Medical College, Manipal, India; ^3^ India Health Fund, Tata Trusts, Mumbai, India; ^4^ Ramniranjan Jhunjhunwala College of Arts, Science, and Commerce, Mumbai, India

## Abstract

**PURPOSE:**

To understand the preferences and attitudes of patients and family caregivers on disclosure of cancer diagnosis and prognosis in an Indian setting.

**METHODS:**

Overall, 250 adult patients with cancer and 250 family caregivers attending the outpatients of a tertiary cancer hospital for the first time were recruited purposively. The mean ages of patients and caregivers were 49.9 years (range, 23-80 years) and 37.9 years (range, 19-67 years), respectively. Separately, they completed prevalidated, close-ended preference questions and were interviewed for open-ended attitude questions.

**RESULTS:**

A total of 250 adult patients (response rate, 47.17% overall, 73.2% in men, and 26.8% in women) and 250 family caregivers (response rate, 40.65% overall, 84.0% in men, and 16.0% in women) participated. Significant differences were observed in the preference to full disclosure of the name of illness between patients (81.2%) and caregivers (34.0%) and with the expected length of survival between patients (72.8%) and caregivers (8.8%; *P* < .001). The patients felt that knowing a diagnosis and prognosis may help them be prepared, plan additional treatment, anticipate complications, and plan for future and family. The caregivers felt that patients knowing a diagnosis and prognosis may negatively affect the future course of illness and cause patients to experience stress, depression, loss of hope, and confidence.

**CONCLUSION:**

Patients with cancer preferred full disclosure of their diagnoses and prognoses, whereas the family caregivers preferred nondisclosure of the same to their patients. This novel information obtained through a large study with varied participants from different parts of the country will help formulate communication strategies for cancer care.

## INTRODUCTION

Cancer-related communication is a complex process and not just the mere transfer of information. It is important to understand the preferences of patients and family caregivers about disclosure of diagnosis and prognosis to avoid demoralization and ensure therapeutic bonding.^1^ Although Western medical practice emphasizes completely truthful disclosure of diagnosis, studies have shown that the majority of physicians working in Southern-European and Asian countries do not agree with that^2^: Only one third of Italian patients were informed about their cancer diagnoses and less than half of them knew about their prognoses^3^; the majority of the Japanese population preferred full disclosure of the diagnosis and preferred only partial disclosure of prognosis, because they feared it would become a self-fulfilling prophecy.^4^ Japanese physicians felt that communication of diagnosis and prognosis should be individualized,^5^ and the Tanzanian physicians preferred a reflective approach for disclosure.^6^ Adverse outcomes of disclosure have been seen in some studies involving patients with GI and lung cancers, in which reported pain scores increased and physical/emotional functioning became poorer after disclosure.^7^ Conversely, an insightful disclosure in patients with breast cancer could reduce long-term emotional distress and improve physical health.^8^ Physicians often ascribe patient and family reluctance to know the truth and the psychological morbidity of truth telling as important barriers for disclosure.^9^ In the Western medical practice, the disclosure of a terminal prognosis is justified ethically, because it upholds the principle of self-determination and enables patients to make treatment decisions consistent with their life goals.^10^ However, this does not necessarily apply to the Asian population, for whom autonomy is considered more collective than individual.^11^ In India, patients often are kept in the dark about their health information; a unique form of collusion exists between the oncologist and the family, and the patient, though aware, surprisingly accepts that situation.^12^ There are few big Indian studies that have investigated communication in cancer care, and none have focused on preferences and attitudes of patients and family caregivers on disclosure of cancer diagnosis and prognosis. Such an important but less investigated aspect of care inspired us to embark on this research.

## METHODS

### Study Design and Setting

This was a cross-sectional observational study. It was conducted between February 2017 and August 2017 at a tertiary cancer care center in India.

### Participants

All of the participants recruited for the study had newly diagnosed cancer; they and their family caregivers were attending the oncology outpatient service for the first time. All of the potential participants were identified at the new patient registration counter. Posters were used to solicit the participation of prospective research participants for the study. Due diligence was taken to ensure that the procedure for recruiting participants was not coercive. All patients who were men or women; were older than age 18 years; had an Eastern Cooperative Oncology Group score of 0-213; were able to understand English, Hindi, or Marathi; and were willing to participate in the study were considered eligible patient participants. All caregivers who were men or women; older than age 18 years; able to understand English, Hindi, or Marathi; and willing to participate in the study were considered eligible caregiver participants. Patients and caregivers already counseled about the diagnosis and prognosis or those who have already received cancer-directed treatment before this episode were excluded from the study. All eligible participants received study-related information in the language of their preference. Research assistants administering the questionnaire and conducting the interviews were clinical psychologists who had no role in patient’s current or future disease-related treatment. Written informed consent was taken from all of the participants, and the research assistants answered participant’s queries before the consenting process. All participants were assured that the completed questionnaire would be anonymized and that confidentiality of the individual participants would be maintained.

### Variables

The primary objective was measured using a validated, interviewer-administered questionnaire. The preference questionnaire had seven diagnoses- and four prognosis-related questions and was adapted from previous similar studies.^14,15^ The secondary objective was explored by asking five open-ended attitude questions (Data Supplement). The survey questionnaire was piloted, and content was validated before administering. Patients and family caregivers received different sets of preference questions and were asked different attitude questions. The patients and family caregivers completed the survey questionnaire only once.

### Data Sources/Measurement

Two research assistants recruited the participants. They used a purposive sampling method from various oncology outpatient clinics at the study setting.^16,17^

### Bias

To minimize confounding bias that could develop from family dynamics, we tried to recruit participants from separate family units. Adequate time was given to the participants to minimize response bias.^18^

### Study Size

In the year 2015, there were 37,371 new patients with cancer who sought outpatient oncology consultation in the hospital.^19^ Therefore, for this survey, the sample size of adult patients and caregivers was estimated at 250 each (± 6% margin of error at a 95% CI). The lesser margin of error of ± 5% was not considered because of an enormous increase in sample size. Moreover, the 6% margin of error is acceptable for a survey study with a large patient population.

### Statistical Method and Qualitative Analysis Strategy

The participant’s responses for the preference questions were recorded through quantitative analysis of the close-ended questionnaire administered. Demographics and clinical details were evaluated from the case record forms by descriptive statistics. The χ^2^ test was used for comparison of nominal data, and factors affecting responses were analyzed using multinomial logistic regression analyses. Additional verification was done by assigning ordinal values to the responses (0 = nondisclosure, 0.5 = partial disclosure, 1 = full disclosure) through an ordinal regression. All analyses were done using SPSS software version 24 (SPSS; IBM, Armonk, NY), and a *P* value of < .05 was considered statistically significant. The transcript of responses to attitude questions was content analyzed using NVivo software (QSR International, Victoria Australia)^20^; the responses were recorded as percentages, and themes relevant to each question were generated.^21^ The study was approved by the institutional ethics committee (project No. 1611, dated July 8, 2016), and the study is registered with the Clinical Trials Registry–India (reference No. 2017/10/010138).

## RESULTS

**FIG 1:**
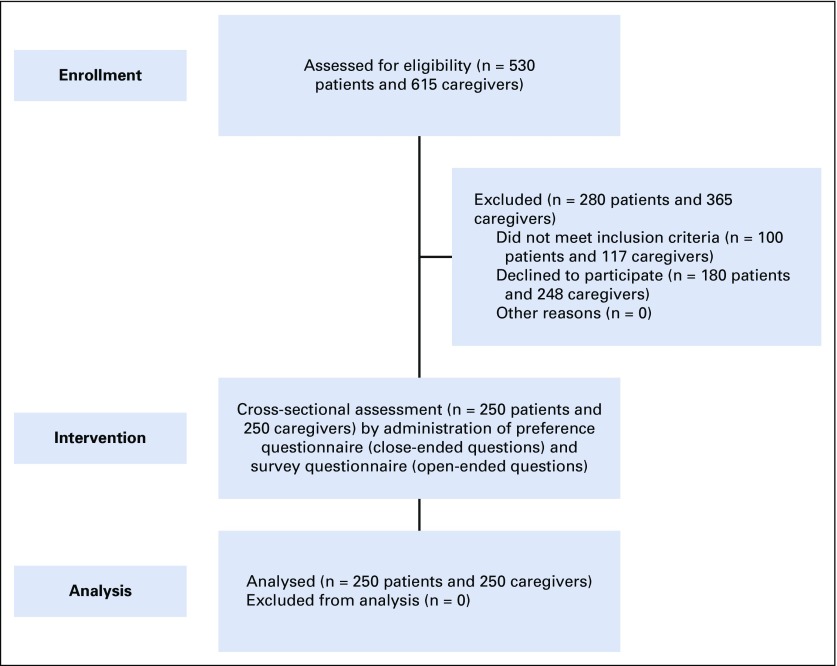
CONSORT 2010 flow diagram.

Among 530 patients and 615 caregivers approached, 250 patients (47.17%) and 250 caregivers (40.65%) participated in this study. Reasons for nonparticipation are summarized in Figure 1. The mean ages of patients and caregivers were 49.9 years (range, 23-80 years) and 37.9 years (range, 19-67 years), respectively. Overall, 73% of the patients and 84% of family caregivers were men, and the majority in each group lived in a semiurban setting (66.4% and 55.6%, respectively). In addition, 90.8% of patients and 74.4% of caregivers were married, and 61.2% and 63.6%, respectively, had nuclear families. The majority of the participants in the patient and caregiver groups had completed secondary (46.8% and 45.2%) or higher (29.2% and 50.8%) education. In 41.6% of patients, the son was the primary caregiver. Head and neck cancer was the most common type of cancer (33.2%), and only 25.2% of patients had another comorbid illness (Table 1).

**TABLE 1:**
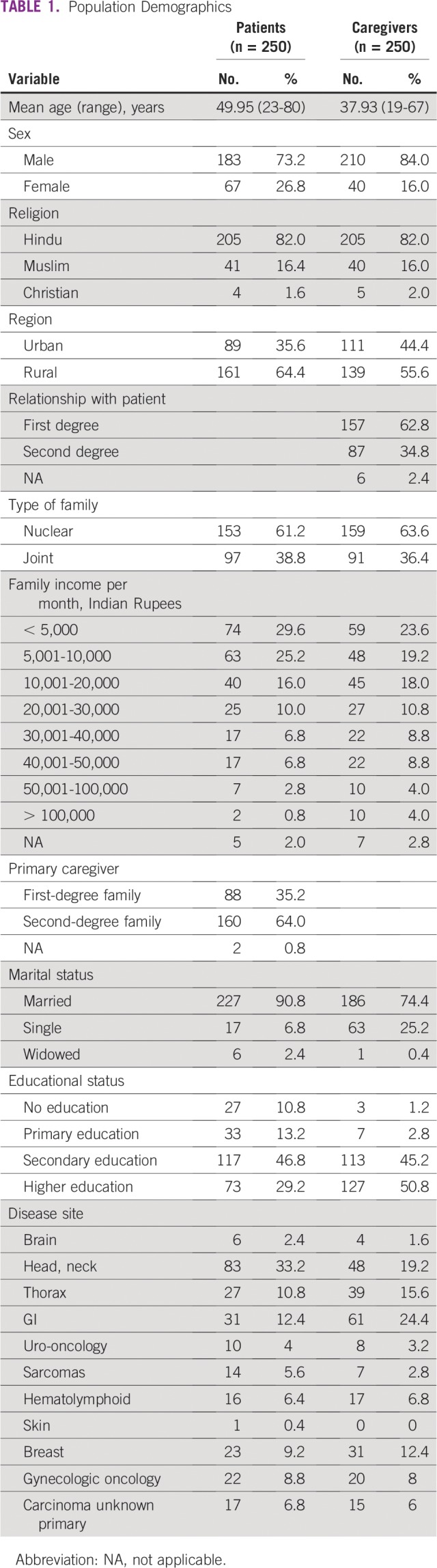
Population Demographics

### Knowing the Name and Seriousness of Illness

In total, 81.2% and 87.2% of patients preferred full disclosure of name and seriousness of the illness, respectively, whereas 34.0% and 26.8% of caregivers preferred full disclosure of diagnosis and seriousness of illness to their patients, respectively. Comparison of preference responses of patients and caregivers showed statistically significant differences (Table 2). The content analysis of the responses to attitude questions showed that 68% of patients felt that knowing the name and seriousness of the illness may positively affect the course of illness. They felt that it was important to know, because it would help them plan additional treatment. However, 32% of the patients felt that this knowledge could negatively affect the course of illness. They also felt that it could cause increased stress, worry, sadness, and depression. A total of 72.4% of the caregivers felt that the patient knowing the name and seriousness of illness may negatively affect the future course of illness. They felt that the patient would be stressed and worried, could lose confidence, and could experience a negative impact on future treatment (Table 3). Comparison of responses to preference and attitude questions by patients and caregivers about patients knowing the name and seriousness of illness was not very different.

**TABLE 2:**
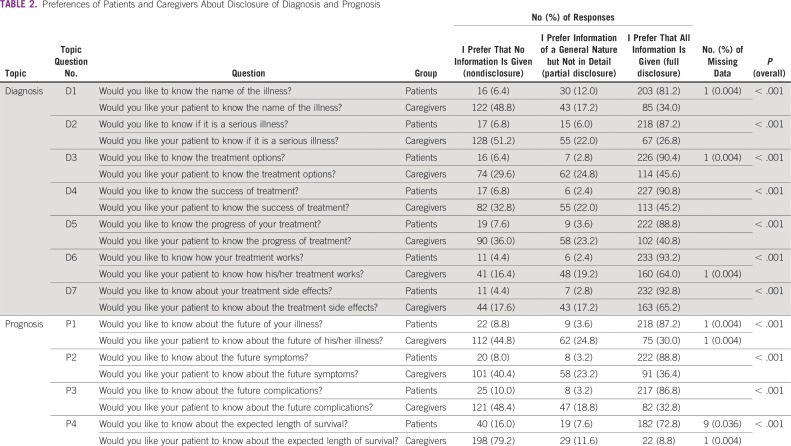
Preferences of Patients and Caregivers About Disclosure of Diagnosis and Prognosis

**TABLE 3:**
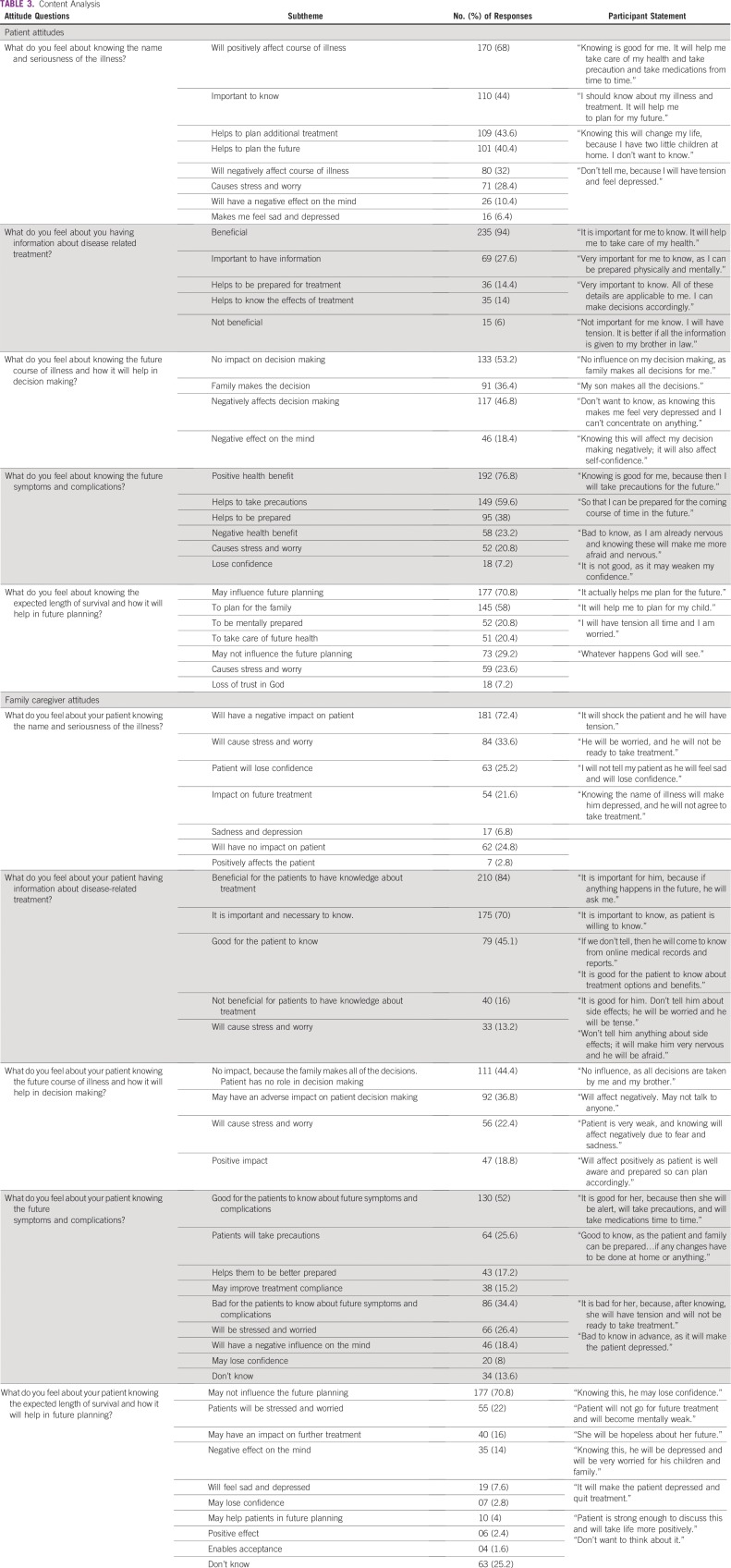
Content Analysis

### Knowing Disease-Related Treatment Information

A total of 90.4% of patients preferred full disclosure of the treatment options; 90.8%, success of treatment; 88.8%, progress of treatment; 93.2%, how the treatment works; and 92.8%, adverse effects of treatment. Among the caregivers, 45.6% of caregivers preferred full disclosure of treatment options to the patient; 45.2%, success of treatment; 40.8%, progress of treatment; 64%, how the treatment works; and 65.2%, adverse effects of treatment. Comparison of responses of patient and caregiver preferences showed statistically significant differences (Table 2). The content analysis of the responses to attitude questions showed that 94% of patients felt that knowing the treatment-related information was beneficial. They felt that knowing the treatment-related information and its effects was important, because it would help them to plan their futures better. In total, 84% of the caregivers also felt that it was beneficial and important for the patients to have treatment-related information (Table 3). Comparison of responses to preference and attitude questions by the patient and caregivers about the patients knowing disease-related treatment showed coherence in patient responses. Although caregivers felt that it was beneficial for the patients to have information about disease-related treatment, they did not agree with disclosing information to patients about treatment options, success of treatment, and progress of treatment. However, they favored patients knowing about how the treatment works and the adverse effects of treatment.

### Knowing the Future Course of Illness

A total of 87.2% of patients preferred full disclosure of the future course of illness. Only 30% of caregivers preferred that their patients should know this information (Table 2). The content analysis of the responses to attitude questions showed that 53.2% of patients felt that knowing the future course of illness has no impact on future decision making, because the family makes all of the decisions. In addition, 46.8% of the participants felt that knowing the future course of illness may negatively affect future decision making. Among the caregivers, 44.4% of caregivers felt that the patient knowing the future course of illness would have no impact on future decision making, because the patient has no role in decision making. In addition, 36.8% caregivers felt that the patient knowing the future course of illness may adversely affect decision making (Table 3). Comparison of responses to preference and attitude questions by the patient and caregivers about patients knowing the future course of illness showed coherence in caregiver responses. Although patients preferred full disclosure of the future course of illness, when they were asked about how this information would affect future decision making, the responses were no impact or negative impact, because families were making decisions for one third to one half of patients.

### Knowing Future Symptoms and Complications

Overall, 88.8% of patients preferred full disclosure of the future symptoms, and 86.8% of patients preferred full disclosure of the future complications. Among the caregivers, only 36.4% preferred full disclosure of patient knowing future symptoms, and 32.8% preferred full disclosure of patients knowing future complications (Table 2). The content analysis of the responses to attitude questions showed that 76.8% of patients felt that knowing future symptoms and complications would have a positive health benefit. They felt that it would help them be prepared and take precautions. Among caregivers, 52% of the caregivers felt that patients knowing about the future symptoms and complications would have a positive health benefit. It could help patients be prepared and take precautions, and it may improve treatment compliance (Table 3). Comparison of responses to preference and attitude questions by the patient and caregivers about the patients knowing future illness and complications showed coherence in patient responses. Although half of the caregivers felt that the patient knowing about future illness and complications would have a health benefit, only one third of caregivers preferred full disclosure.

### Knowing Expected Length of Survival

A total of 72.8% of patients preferred full disclosure of the expected length of survival. Conversely, only 8.8% of caregivers preferred that their patients should know of the expected length of survival (Table 2). The content analysis of the responses to attitude questions showed that 70.8% of patients felt that knowing the expected length of survival may influence future planning. They felt that knowing this information would help them be mentally prepared, plan for the family, and take care of future health. Among the caregivers, 70.8% of the caregivers felt that knowing the expected length of survival may not influence future planning. They felt that the patient knowing the expected length of survival could cause stress and worry to the patients, have a negative impact on future treatment, and cause sadness and loss of confidence (Table 3). Comparison of responses to preference and attitude questions by the patient and caregivers on patients knowing the expected length of survival were coherent.

### Factors Influencing Patient and Caregiver Preferences

Multinomial regression of factors showed that patients with postgraduate qualifications preferred full disclosure of the progress of treatment and future symptoms. Among caregivers, married female caregivers preferred nondisclosure or partial disclosure in most of the items of the preference questionnaire. Caregivers from the Eastern states of India, those with a higher secondary-level education, and those caring for patients with genitourinary cancers preferred nondisclosure or partial disclosure in some items of the preference questionnaire (Table 4). Ordinal regression did not supply any different results (Data Supplement).

**TABLE 4:**
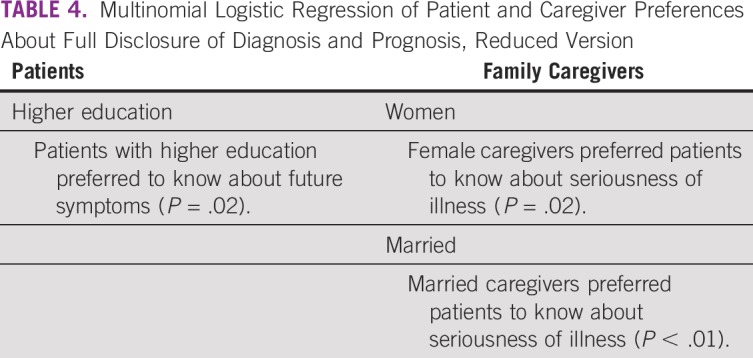
Multinomial Logistic Regression of Patient and Caregiver Preferences About Full Disclosure of Diagnosis and Prognosis, Reduced Version

## DISCUSSION

In India, there is a common misbelief that the patients need not know much about their illnesses or treatments,^22^ especially with serious illnesses like cancer.^23^ This notion urges the caregivers to try to protect the patients from adverse health information.^15^ The findings from this study contradict such beliefs and provide novel insight into cancer-related communication about disclosure of diagnosis and prognosis.

In this study, patients preferred to know treatment options, successes, and adverse effects—a behavior showing positive trends with education and active decision making. These findings can be corroborated with studies done in Europe14,^2^,^4^,^2^,^5^ and Australia.^26,27^ Also, our patients preferred to know about future symptoms, complications, and expected length of survival—truth-seeking behavior seen elsewhere in communication-studies focusing on prognosis and life expectancy.^28,29^

Nevertheless, it is important to highlight the small percentage of patients who expressed reservations about full disclosure of information. They feared that it could lead to increased stress, anxiety, and depression and could have a negative impact on future treatment choices. Such behavior is not unique to our society and has been seen elsewhere too: Qualitative studies have shown that patients neither preferred to know bad prognoses^30^ nor wanted physicians to be too specific about the prognosis components^9^; rather, they preferred that physicians check with them before disclosing the information,^31^ and they wanted prognostic information to be presented with positively framed language in terms of survival probabilities rather than risk of mortality.^32^ In these studies, participants also felt that having a loved one by their side helped them cope with the information better.^33^ These protective behaviors have a deeper perspective. In traditional societies like India, the family is part of the illness experience, and the disease is perceived as a disease of the family.^34^ Family support helps to share the physical, social, and financial responsibilities and helps patients emotionally cope with the illness experience.^35^ These aspects resonates in this study, in which some of the family caregivers were largely opposed to disclosure of the diagnosis and prognosis to the patients. They felt that patients knowing about the diagnosis and prognosis could cause stress, loss of confidence, and depression and might negatively affect the future course of illness. The families, thus, showed protective behavior to shield their patients from bad news, which in Oriental societies has often been considered an act of love.^36^ In this study, families felt that patients have no role in decision making and that families should make all of the decisions for the patient, as seen in an Egyptian study, in which families felt that the patient must be dependent and nurtured and not involved in the decision making.^37^ Although degree of family involvement varies across the cultures, focusing on family functioning during communication may improve outcomes.^38^

This study is one of largest studies of its kind, covering a cross-section of patients hailing from all over India who were treated at tertiary referral cancer center. To avoid bias in response, all participants received the study questionnaire and were interviewed at the registration desk before meeting the physician. To avoid any cross-transfer of information, questionnaire completion and interviews were conducted separately for the participants.

A purposive sampling brings bias into the study. Although the research site sees patients from across India, it is still a single-center study. Therefore, the perspectives of the research participants may not represent the views of the entire Indian population. The study has an inherent bias, because the research was conducted in a cancer hospital, and the patients may have already had knowledge about their diagnoses. There were situations in which families prevented patients from participating in the study. We assume that those patients were the ones who had no information, or that their families were withholding the information. We were thus unable to know their preferences. We are aware that an interplay of complex cultural, demographic, clinical (eg, cancer stages, sites), nonverbal,^39^ and circumstantial^40^ factors might direct people to have different attitudes and preferences about the disclosure of cancer-related communication^41,42^; it was beyond the scope of this study to delve deeper into these topics from the data we had.

There was discordance between patients and families about preferences and attitudes toward disclosure of diagnosis and prognosis. Patients with cancer preferred full disclosure of their diagnostic and prognostic information, whereas family caregivers preferred nondisclosure of the same. The patients felt that knowing such information may help them to plan additional treatment, anticipate complications, and plan for the future. The caregivers, conversely, felt that letting patients know such information would negatively affect the future course of illness and infuse stress, depression, loss of hope, and confidence in their lives. We feel that effective physician communication may help harmonize these discordances.
